# The OsAP4-OsCATA/OsCATC Regulatory Module Orchestrates Drought Stress Adaptation in Rice Seedlings Through ROS Scavenging

**DOI:** 10.3390/plants14142174

**Published:** 2025-07-14

**Authors:** Yifei Jiang, Bin Xie, Xiong Luo, Yangsheng Li

**Affiliations:** State Key Laboratory of Hybrid Rice, College of Life Sciences, Wuhan University, Wuhan 430072, China; smilexbin@whu.edu.cn (B.X.); 2022202040081@whu.edu.cn (X.L.)

**Keywords:** OsAP4, OsCATs, drought stress, ROS scavenging, rice

## Abstract

Drought stress poses a major constraint on global crop productivity. Although aspartic proteases (APs) are primarily characterized in plant disease resistance, their roles in abiotic stress adaptation remain largely unexplored. Here, we demonstrate that rice (*Oryza sativa*) OsAP4 critically regulates drought stress tolerance at the seedling stage. Genetic manipulation through overexpression (*OsAP4*-OE) or CRISPR knockout (*OsAP4*-KO) resulted in significantly reduced or enhanced stress tolerance compared to wild-type plants, respectively. Through integrated approaches including yeast two-hybrid, bimolecular fluorescence complementation, pull-down, co-immunoprecipitation, and protein degradation assays, we established that OsAP4 physically interacts with and destabilizes OsCATA/OsCATC, two catalase enzymes responsible for reactive oxygen species (ROS) scavenging. Importantly, OsAP4 modulates ROS production under drought stress treatment conditions. Together, these findings reveal a novel OsAP4-OsCATA/OsCATC regulatory module governing rice drought stress responses.

## 1. Introduction

Aspartic proteases (APs) represent a conserved class of intracellular hydrolytic enzymes with a ubiquitous distribution across biological kingdoms. These enzymes have been detected in various plant tissues, such as seeds, leaves, and stems, as well as in microorganisms, fungi, and animal systems [1,2,3]. The catalytic mechanism of APs involves two essential aspartic acid residues that cooperatively mediate water molecule activation for peptide bond cleavage, with most members exhibiting optimal activity under acidic conditions. Structurally, the complete AP domain comprises approximately 330 amino acids containing characteristic Asp-Thr/Ser-Gly residues, while proteins retaining partial domains with functional catalytic residues are classified as atypical APs [4]. The remarkable sequence diversity within the AP family underlies their functional specialization, enabling participation in diverse biological processes, including photomorphogenesis through light signaling pathways, innate and adaptive immune responses, and precise proteolytic processing of precursor proteins [5,6,7].

Plant APs play pivotal roles in regulating diverse physiological processes. In plant–pathogen interactions, several APs exhibit antimicrobial functions: *Arabidopsis* SAP1 and SAP2 suppress the growth of *Pseudomonas syringae* [8], lychee LcAP1 counteracts PlPeL8-induced pathogenic infection [9], and soybean GmAP5 confers resistance against *Phytophthora sojae* [10]. Regarding growth regulation, *Arabidopsis* ECS1 and ECS2 mediate sperm–egg cell fusion [11], wheat APP-A1 modulates grain morphology and yield [12], poplar AP17 and AP45 influence xylem development [13], while cotton GhAP3 regulates fiber elongation [14]. Under abiotic stress conditions, *Arabidopsis* APA1 and ASPG1 are associated with drought tolerance, while pineapple AcAP1 is linked to cold tolerance [15,16,17].

The rice genome harbors approximately 100 AP genes distributed across its 12 chromosomes [18], yet their functional characterization remains incomplete. Current research has identified several OsAPs with specialized roles: OsAsp1 and OsAP65 regulate organ development [19], while OsAP25 and OsAP37 mediate programmed cell death [20,21]. In plant immunity, distinct OsAPs exhibit pathogen-specific defense functions—OsAP47 confers resistance to rice black-streaked dwarf virus [22], OsAP77 enhances blast resistance [23], and OsCDR1 protects against bacterial blight [24]. The S5 protein stands out as a particularly valuable genetic resource for hybrid breeding due to its wide-compatibility traits [25]. While these findings highlight the functional versatility of OsAPs in development and biotic stress responses, critical knowledge gaps persist. Most notably, their potential roles in abiotic stress adaptation and corresponding molecular mechanisms remain largely unexplored.

Previous studies suggested OsAP4’s potential role in rice yield regulation [26], whereas our phenotypic evaluation revealed its limited impact on yield traits. Consequently, leveraging our laboratory’s expertise in abiotic stress responses, we focused on characterizing OsAP4’s functions under stress conditions. Here, we demonstrate that the rice aspartic protease OsAP4 negatively modulates drought stress tolerance. OsAP4 reduces the stability of ROS-scavenging enzymes, leading to impaired ROS homeostasis under stress conditions and ultimately attenuating plant drought tolerance. These findings advance our understanding of drought adaptation mechanisms in crops.

## 2. Materials and Methods

### 2.1. Bioinformatic Analysis and Statistical Analysis

Evolutionary relationships were analyzed by constructing phylogenetic trees with TBtools software (version 2.315) [27]. Promoter regulatory elements were predicted using the PlantCARE database (version 1.0) [28]. All statistical comparisons were generated using GraphPad Prism (version 8.0).

### 2.2. Plant Materials, Drought Stress Treatment, and Phenotypic Measurements

For overexpression studies, the full-length *OsAP4* CDS from the *indica* rice variety 9311 was inserted into the *pCAMBIA1301* vector downstream of the constitutive *CaMV35S* promoter. The construct was introduced into 9311 plants through *Agrobacterium*-mediated transformation (Biorun, Wuhan, China). For loss-of-function analysis, we created *OsAP4* knockout mutants in the 9311 background using CRISPR-Cas9 genome editing. Additionally, *OsCATA* and *OsCATC* mutant lines in the ZH8015 genetic background were acquired from previously published work [29].

For the drought stress tolerance experiment, we employed a 25% PEG6000 (*w*/*v*) treatment to simulate drought conditions. The experimental procedure was as follows: Germinated rice seeds were initially cultivated in standard Yoshida nutrient solution. Two-week-old seedlings were then transferred to Yoshida solution supplemented with 25% PEG6000 for stress treatment. Overexpression and knockout lines were subjected to treatment for 3 and 5 days, respectively, before being returned to normal Yoshida solution for recovery. All plants were maintained in a controlled greenhouse environment at 30 °C with a 10 h light/14 h dark photoperiod. Following a 6-day recovery period, we measured seedling height (H0 for control, H1 for treatment) and fresh weight (W0 for control, W1 for treatment). Relative seedling height and relative biomass were calculated as H1/H0 and W1/W0, respectively. Survival rates were determined after a 10-day recovery period. For each parameter (relative plant height, relative biomass, and survival rate), we conducted a minimum of five biological replicates, with each replicate containing at least 20 seedlings to ensure statistical reliability.

For additional treatments, submergence stress was achieved by fully immersing plants in deionized water. Saline stress was applied by growing seedlings in Yoshida nutrient solution containing 100 mM NaCl. Heat stress was induced by incubating plants at 42 °C in a temperature-controlled growth chamber.

### 2.3. Measurement of Physiological and Biochemical Indices

Approximately 100 mg of fresh leaves per sample were collected from the transgenic lines and the wild-type before and after cold treatment. We performed physiological and biochemical analyses according to the manufacturers’ protocols, including 3,3′-diaminobenzidine (DAB) staining (US EVERBRIGHT, Suzhou, China), ferric-reducing antioxidant power (FRAP), catalase (CAT) activity, and malondialdehyde (MDA) content (Solarbio, Beijing, China). Three biological replicates were performed.

### 2.4. Transcript Level Analysis

The experimental procedure for RT-qPCR analysis was as follows: Total RNA was extracted from plant leaves using RNA-Easy Isolation Reagent (Vazyme, Nanjing, China), followed by cDNA synthesized with HiScript IV RT SuperMix for qPCR (+gDNA wiper; Vazyme). Quantitative PCR was conducted using Hieff UNICON^®^ Universal Blue qPCR SYBR Green Master Mix (Yeasen, Shanghai, China), with *OsActin* serving as the internal control. Transcript-level expression analysis was complemented by RNA-seq data from the TENOR database [30]. All experiments included three biological replicates, and the primers used are shown in Table S1.

### 2.5. Subcellular Localization

For the subcellular localization assay, we adopted an established protocol [31]. Briefly, the coding sequences of *OsAP4*, *OsCATA*, and *OsCATC* from *indica* rice cultivar 9311 were PCR-amplified and individually fused to GFP in the *pBWA(V)HS-osgfp* vector. The resulting constructs (*OsAP4-GFP*, *OsCATA-GFP*, and *OsCATC-GFP*) were co-transformed with the nuclear marker *H2B-RFP* into *Agrobacterium* tumefaciens strain GV3101. Transient expression was achieved through leaf infiltration of 4-week-old *N. benthamiana* plants. Following 48 h incubation in darkness at 22 °C, subcellular localization patterns were examined using a Leica SP8 confocal microscope (Leica Microsystems, Wetzlar, Germany). The primers used are shown in Table S1.

### 2.6. Yeast Two-Hybrid (Y2H) Assay

For the Y2H assay, the full-length coding sequences of *OsAP4* and *OsCATA/OsCATC* were amplified and directionally cloned into *pGBKT7* (bait vector) and *pGADT7* (prey vector), generating *BD-OsAP4*, *AD-OsCATA*, and *AD-OsCATC* constructs, respectively. The protein–protein interaction pairs (*BD-OsAP4* × *AD-OsCATA* and *BD-OsAP4* × *AD-OsCATC*) were co-transformed into *Saccharomyces* cerevisiae strain AH109 using the lithium acetate method. Concurrent transformations were performed on the positive control (*BD-53 × AD-T*) and negative control (*BD-Lam × AD-T*). Transformants were selected on SD/-Leu/-Trp dropout medium and subsequently screened on SD/-Ade/-His/-Leu/-Trp/X-α-Gal plates (Coolaber, Beijing, China) according to the manufacturer’s protocol. The primers used are shown in Table S1.

### 2.7. Bimolecular Fluorescence Complementation (BiFC) Assay

For the BiFC assay, the full-length coding sequences of *OsAP4* and *OsCATA*/*OsCATC* were cloned into *pXY106* (YFPN fragment) and *pXY104* (YFPC fragment) vectors, generating *OsAP4-YFPN*, *OsCATA-YFPC*, and *OsCATC-YFPC* fusion constructs, respectively. The interaction pairs (*OsAP4-YFPN × OsCATA-YFPC* and *OsAP4-YFPN × OsCATC-YFPC*) were co-transformed into *Agrobacterium* tumefaciens strain GV3101. After injection into *N. benthamiana* leaves and 48 h of incubation, fluorescence signals were visualized using a Leica SP8 confocal microscope. Positive protein–protein interactions were indicated by reconstituted YFP fluorescence in the epidermal cells of infiltrated leaves. The primers used are shown in Table S1.

### 2.8. Pull-Down Assay

For the pull-down assay, the full-length coding sequences of *OsAP4* and *OsCATA*/*OsCATC* were cloned into *pET30a-His* and *pGEX-6P-1* expression vectors, generating *OsAP4-His*, *OsCATA-GST*, and *OsCATC-GST* fusion constructs, respectively. These recombinant plasmids were transformed into *E. coli* BL21 (DE3) competent cells for protein expression. The purified OsAP4-His protein was incubated with His-tagged magnetic beads along with either OsCATA-GST or OsCATC-GST at 4 °C overnight. Following incubation, the beads were washed three times with PBS buffer, resuspended in 1 × SDS-PAGE loading buffer, and boiled for 5 min. Protein interactions were subsequently analyzed via immunoblotting using anti-GST and anti-His antibodies. The primers used are shown in Table S1.

### 2.9. Co-Immunoprecipitation (Co-IP) Assay

For the Co-IP assay, the full-length coding sequences of *OsAP4* and *OsCATA*/*OsCATC* were inserted into *Pan580-HA* and *pBWA(V)Hs-MYC* vectors, generating *OsAP4-HA*, *OsCATA-MYC*, and *OsCATC-MYC* fusion constructs, respectively. The *OsAP4-HA* construct was co-expressed with either *OsCATA-MYC* or *OsCATC-MYC* in *Agrobacterium* tumefaciens strain GV3101, which was then used to infiltrate *N*. *benthamiana* leaves. Total proteins extracted from the infiltrated leaves were incubated with MYC-tagged magnetic beads at 4 °C overnight. After three washes with PBS buffer, the bound proteins were eluted by boiling in 1 × SDS-PAGE loading buffer, separated by SDS-PAGE, and subsequently analyzed via immunoblotting using anti-MYC and anti-HA antibodies. The primers used are shown in Table S1.

### 2.10. In Vivo Protein Degradation Assay

Protein degradation assays were performed according to an established protocol [32]. *Agrobacterium* strains harboring both *OsAP4-HA* and either *OsCATA-MYC* or *OsCATC-MYC* were co-infiltrated into *N. benthamiana* leaves, with control groups expressing *OsCATA-MYC* or *OsCATC-MYC* alone, respectively. Following a 3 h drought stress treatment, leaf samples were collected for total protein extraction. Protein concentrations were normalized prior to analysis. Degradation was monitored via immunoblotting using an anti-MYC antibody.

## 3. Results

### 3.1. Expression Patterns of OsAP4

**Figure 1:**
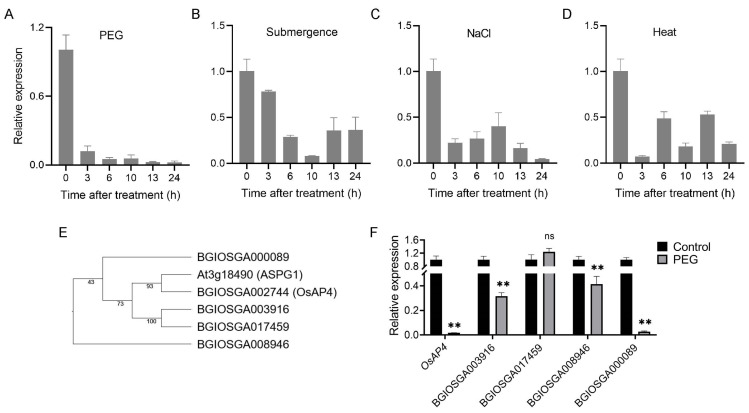
Expression of *OsAP4* under abiotic stress conditions. (**A**–**D**) *OsAP4* expression under PEG, submergence, NaCl, and heat stress treatments from 0–24 h. Data are means ± SD (*n* = 3). (**E**) Evolutionary relationships of ASPG1 and five homologous aspartic proteinase family members of rice. (**F**) Expression of the five homologues under control and PEG stress treatment conditions. Data are means ± SD (*n* = 3); ns, not significant; ** *p* < 0.01 determined by *t*-test; the test was performed on samples between the control and PEG treatment.

OsAP4, a member of the AP family in rice, was investigated for its response to abiotic stresses. Two-week-old seedlings of the *indica* rice variety 9311 were subjected to four treatments: PEG (drought mimic), submergence, NaCl, and heat for 0–24 h. RT-qPCR analysis showed that all treatments suppressed *OsAP4* expression, with PEG inducing the most drastic and consistent suppression—a 43-fold reduction at 24 h (Figure 1A–D). Among the five rice AP family homologs of *Arabidopsis* drought-regulated *ASPG1*, *OsAP4* exhibited the strongest response to PEG treatment (Figure 1E,F). Additional drought-mimic treatments confirmed this pattern (Figure S1). Haplotype analysis of *OsAP4* using the MBKbase database [33] revealed that germplasms (*n* ≥ 10) carrying *Hap 3* showed significantly higher drought tolerance than those with *Hap 1/5* (Figure S2). These results suggest OsAP4’s functional importance in drought response.

### 3.2. OsAP4 Negatively Regulates Drought Stress Tolerance in Rice

**Figure 2:**
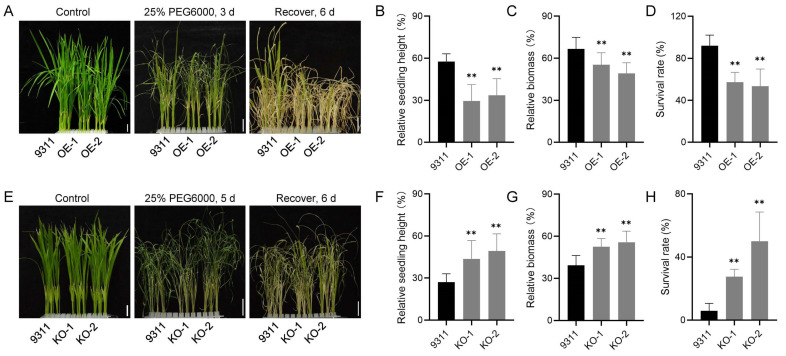
Analysis of drought stress tolerance in *OsAP4* transgenic plants. (**A**) Plant phenotypes, (**B**) relative seedling height, (**C**) relative biomass, and (**D**) survival rates of wild-type 9311 and *OsAP4* overexpression lines under PEG-treated conditions. (**E**) Plant phenotypes, (**F**) relative seedling height, (**G**) relative biomass, and (**H**) survival rates of 9311 and *OsAP4* knockout lines under PEG-treated conditions. In (**B**–**D**,**F**–**H**), data are means ± SD (*n* ≥ 5); ** *p* < 0.01 determined by *t*-test; the test was performed on samples between the 9311 and OE/KO lines. Bars = 2 cm in (**A**,**E**).

To explore the biological function of OsAP4, we constructed overexpression and knockout lines of *OsAP4*. Compared with the wild-type 9311, the transcriptional level of *OsAP4* was significantly upregulated in the OE lines. In contrast, the KO lines exhibited a frameshift mutation due to base insertion, resulting in premature translation termination (Figure S3). Homozygous *OsAP4* transgenic plants were exposed to 25% PEG6000 for drought stress tolerance studies. The relative seedling height and relative biomass can reflect the growth rate of plants, and both metrics were found to be significantly lower in the overexpression lines than in 9311 after recovery (Figure 2A–C). We also investigated the survival rate and found that the survival rate of overexpression lines ranged from 28.6–71.4%, whereas that of 9311 was above 90.0% (Figure 2D). In contrast, the knockout lines showed a higher relative seedling height and relative biomass compared to 9311 after recovery (Figure 2E–G). In terms of survival performance, the survival rate of the knockout lines was about 21.7–72.7%, while that of 9311 was less than 6.0% (Figure 2H). Together, these data confirm that overexpression of *OsAP4* reduces drought stress tolerance, whereas knockout of *OsAP4* increases drought stress tolerance in rice.

### 3.3. Identification of Proteins Interacting with OsAP4

**Figure 3:**
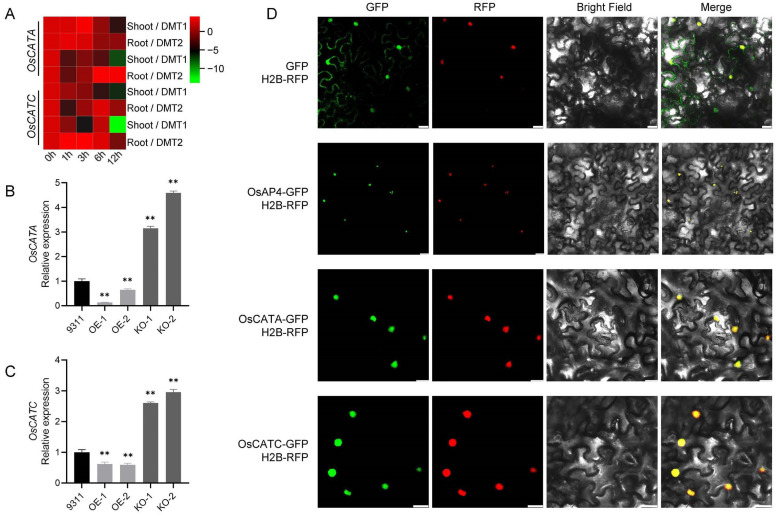
Analysis of candidate OsAP4-interacting proteins. (**A**) Expression of *OsCATA*/*OsCATC* in shoot and root of the plants under drought-mimicking type (DMT) from 0 to 12 h. (**B**,**C**) Expression of *OsCATA/OsCATC* in 9311 and *OsAP4* transgenic plants under PEG treatment conditions. Data are means ± SD (*n* = 3); ** *p* < 0.01 determined by *t*-test; the test was performed on samples between the 9311 and OE/KO lines. (**D**) Subcellular localization of OsAP4 and OsCATA/OsCATC in *N. benthamiana* leaf cells. Bars = 20 μm.

To identify OsAP4-interacting proteins, total proteins from rice plants were incubated with *E. coli*-expressed OsAP4-His for pull-down assays coupled with mass spectrometry. Database annotation revealed two candidate catalase isoforms—OsCATA and OsCATC—as potential interactors. Both simulated drought treatments induced significant alterations in the expression levels of *OsCATA* and *OsCATC* in either shoot or root tissues (Figure 3A; Table S2). Notably, opposite expression patterns of both genes emerged in *OsAP4* overexpression versus knockout lines after drought treatment (Figure 3B,C). Our results corroborate previous findings demonstrating nuclear localization of OsCATA and OsCATC in *N. benthamiana* [29], confirming their predominant nuclear accumulation. Significantly, subcellular localization analysis revealed that OsAP4 exhibits identical nuclear targeting patterns to both OsCATA and OsCATC (Figure 3D). Collectively, these findings support the identification of OsCATA and OsCATC as candidate interacting partners of OsAP4.

### 3.4. OsAP4 Interacts with and Destabilizes OsCATA/OsCATC

**Figure 4:**
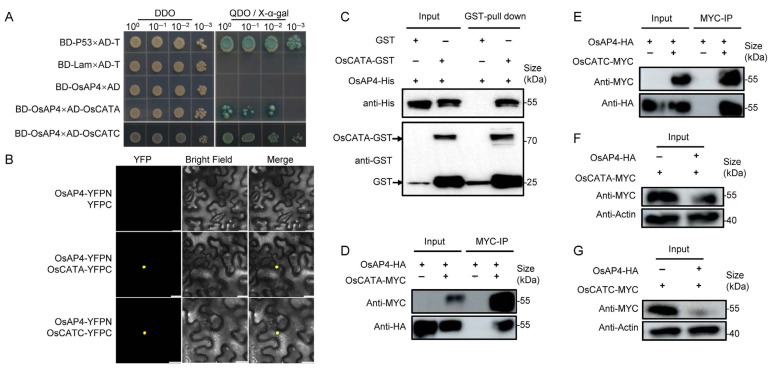
OsAP4 interacts with OsCATA/OsCATC and reduces their stability. (**A**) Validation of OsAP4 interactions with OsCATA/OsCATC by Y2H assay. (**B**) Validation of OsAP4 interactions with OsCATA/OsCATC by BiFC assay. Bars = 20 μm. (**C**) Validation of OsAP4 interactions with OsCATA by pull-down assay. (**D**,**E**) Validation of OsAP4 interactions with OsCATA/OsCATC by Co-IP assay. (**F**,**G**) Validation of OsAP4 mediates OsCATA/OsCATC stability.

The rice CAT family consists of three members: OsCATA, OsCATB, and OsCATC. To confirm whether OsAP4 interacts with OsCATs, we initially performed Y2H experiments, which revealed specific interactions between OsAP4 (BD-OsAP4) and OsCATA/OsCATC (AD-OsCATA/AD-OsCATC), evidenced by colony growth on selective medium (Figure 4A). As the Y2H assays failed to detect interaction between OsAP4 and OsCATB, we focused subsequent validation on the observed interactions with OsCATA and OsCATC through BiFC analysis. The BiFC analysis revealed strong YFP fluorescence signals in the nucleus when OsAP4-YFPN was co-expressed with either OsCATA-YFPC or OsCATC-YFPC (Figure 4B). Pull-down assays using purified OsAP4-His and OsCATA-GST/OsCATC-GST proteins demonstrated specific binding between OsAP4-His and OsCATA-GST, but not with OsCATC-GST or GST alone (Figure 4C). In addition, Co-IP experiments confirmed that OsAP4-HA was immunoprecipitated with both OsCATA-MYC and OsCATC-MYC (Figure 4D,E). To examine the regulatory role of OsAP4 in modulating OsCATA/OsCATC protein stability, we performed transient co-expression assays in *N. benthamiana*. Western blot analysis showed that OsAP4-HA expression decreased the protein levels of both OsCATA-MYC and OsCATC-MYC compared to controls (Figure 4F,G). Overall, these results demonstrate that OsAP4 physically interacts with OsCATA/OsCATC and negatively regulates their protein stability.

### 3.5. OsAP4 Controls ROS Scavenging Capacity

**Figure 5:**
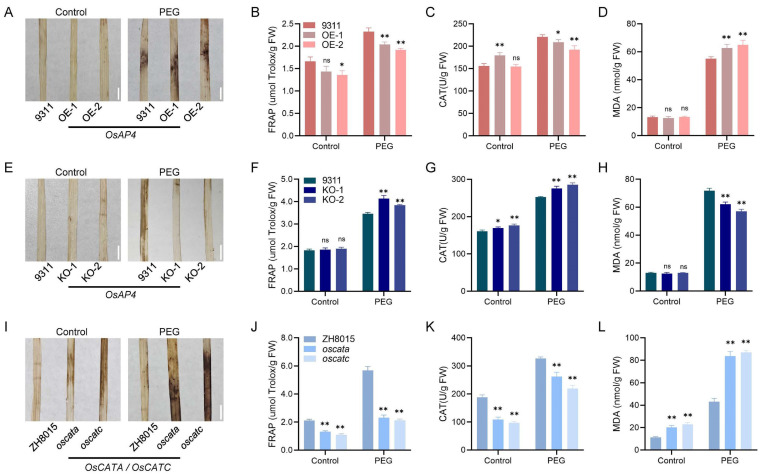
ROS content in *OsAP4* and *OsCATA/OsCATC* transgenic plants. (**A**–**D**) DAB staining, FRAP, CAT activity, and MDA content in leaves of 9311 and *OsAP4* overexpression plants under control and PEG stress conditions. (**E**–**H**) DAB staining, FRAP, CAT activity, and MDA content in leaves of 9311 and *OsAP4* knockout plants under control and PEG stress conditions. (**I**–**L**) DAB staining, FRAP, CAT activity, and MDA content in leaves of ZH8015, *oscata,* and *oscatc* plants under control and PEG stress conditions. In (**B**–**D**,**F**–**H**,**J**–**L**), data are means ± SD (*n* = 3); ns, not significant; * *p* < 0.05, ** *p* < 0.01 determined by *t*-test; the test was performed on samples between the 9311 and OE/KO lines or between the ZH8015 and *oscata*/*oscatc* lines. Bars = 5 mm in (**A**,**E**,**F**).

Catalase, which catalyzes the decomposition of hydrogen peroxide (H_2_O_2_) into water and oxygen, serves as a crucial component in the oxidative defense system of organisms [34]. Given our demonstration of the interaction between OsAP4 and OsCATA/OsCATC, we postulated that OsAP4 might regulate ROS homeostasis. To test this hypothesis, we assessed oxidative damage in drought-stressed *OsAP4* transgenic plants. DAB staining analysis revealed that *OsAP4* overexpression lines accumulated more ROS, while knockout lines exhibited less ROS accumulation compared to wild-type 9311 (Figure 5A,E). Quantitative measurements showed that *OsAP4* overexpression lines had reduced CAT activity, lower FRAP, and elevated MDA content (Figure 5B–D). Conversely, *OsAP4* knockout lines displayed increased CAT activity, higher FRAP, and decreased MDA levels relative to wild-type 9311 (Figure 5F–H). Furthermore, we observed that *OsCATA*/*OsCATC* mutants accumulated more ROS than wild-type ZH8015 under drought stress (Figure 5I–L). These findings collectively demonstrate that OsAP4 cooperates with OsCATA and OsCATC to regulate ROS scavenging capacity during drought stress.

### 3.6. OsAP4 and OsCATA/OsCATC Expression in Response to ABA

**Figure 6:**
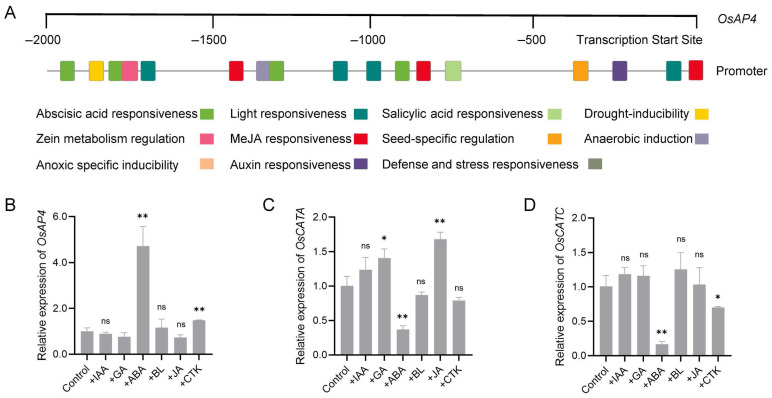
Analysis of *OsAP4* and *OsCATA*/*OsCATC* expression under hormone treatment. (**A**) Analysis of the promoter sequence of *OsAP4*. (**B**–**D**) Expression of *OsAP4*, *OsCATA*, and *OsCATC* in 9311 plants under PEG treatment conditions after being stimulated with IAA, GA, ABA, BL, JA, and CTK hormones. In (**B**–**D**), data are means ± SD (*n* = 3); ns, not significant; * *p* < 0.05, ** *p* < 0.01 determined by *t*-test; the test was performed on samples between the control and IAA/GA/ABA/BL/JA/CTK treatment.

The *Arabidopsis* ASPG1 protein, a functional homolog of OsAP4 (Figure 1E), has been shown to mediate drought responses through the abscisic acid (ABA) signaling pathway [16]. To elucidate the signaling mechanism of OsAP4 in rice under drought stress, we performed a promoter analysis of *OsAP4* and identified multiple hormone-responsive cis-elements within the 2 kb upstream region, including ABA, salicylic acid (SA), jasmonic acid (JA), and auxin response elements (Figure 6A; Table S3). To functionally characterize these regulatory elements, we treated drought-stressed 9311 seedlings with 50 μM solutions of various phytohormones, including indole-3-acetic acid (IAA), gibberellin (GA), ABA, brassinolide (BL), JA, and cytokinin (CTK). RT-qPCR analysis revealed that *OsAP4* expression was most strongly induced by ABA, with CTK showing secondary induction (Figure 6B). Notably, *OsCATA* and *OsCATC* exhibited their most pronounced differential expression patterns in response to ABA treatment (Figure 6C,D), collectively suggesting that ABA may serve as a key regulator of the OsAP4-OsCATA/OsCATC module during drought stress responses.

## 4. Discussion

In the context of global climate change characterized by rising temperatures, declining soil moisture, and escalating agricultural water demands, drought stress has emerged as a major threat to plant productivity. Understanding the molecular mechanisms of drought adaptation in crops is crucial for developing sustainable agricultural strategies. This study identified and functionally characterized *OsAP4*, an AP-encoding gene in rice. We demonstrate that OsAP4 coordinates with OsCATs to orchestrate ROS homeostasis and mediate drought stress signaling pathways during early seedling development, expanding the molecular regulatory network underlying drought stress adaptation in rice.

Drought stress severely impairs plant growth and development, as evidenced by previous studies showing inhibited cotton root system development and reduced photosynthate accumulation in rice under water deficit conditions [35,36]. Interestingly, genetic approaches like *SINAL7* overexpression have demonstrated the potential to enhance *Arabidopsis* drought tolerance by increasing biomass [37]. In our investigation, drought stress markedly slowed seedling growth and induced severe leaf wilting in rice (Figure 2A,E), confirming its detrimental effects on plant development. Following post-drought recovery, phenotypic analysis revealed contrasting growth patterns among the transgenic lines: *OsAP4* overexpression lines had a reduced relative seedling height, while knockout lines showed an increased relative seedling height (Figure 2B,F). Biomass measurements confirmed this inverse relationship (Figure 2C,G). These results demonstrate that OsAP4 may function as a negative regulator of biomass accumulation during drought adaptation in rice, presenting an opposing mechanism to positive regulators such as SINAL7. Importantly, OsAP4 likely plays a pivotal role in balancing the trade-off between stress tolerance and growth development in plants.

ROS act as crucial signaling molecules during plant drought responses. Previous studies have established that various genes modulate drought tolerance through ROS regulation: *MdMRLK2* in apple enhances antioxidant enzyme activities [38], *ZmSRO1d* in maize mediates stomatal ROS production [39], and *OsMRLK63* in rice interacts with NADPH oxidases to promote ROS generation [40]. Our study reveals that OsAP4 physically interacts with catalases OsCATA/OsCATC (Figure 4A–E), known ROS-scavenging enzymes whose overexpression enhances drought resistance [41]. Further, we observed an inverse correlation between *OsAP4* expression and drought tolerance—overexpression lines showed elevated ROS accumulation and reduced stress resistance, while knockout lines displayed lower ROS levels and improved tolerance (Figure 5A–H). These findings establish that OsAP4 negatively regulates drought resistance in rice seedlings through modulation of the ROS signaling pathway. Given the pleiotropic roles of ROS in stress responses, we hypothesize that the OsAP4-OsCATA/OsCATC module may represent a convergent node for multiple stress signaling pathways, though this requires experimental validation.

Plant hormones play pivotal regulatory roles in various physiological processes, with ABA being particularly crucial for drought responses. Numerous studies have established that drought stress induces ABA accumulation, and several key regulators of the ABA signaling pathway—including maize ZmbHLH105, wheat TaFDL2-1A, and rice OsNAC120—have been identified [42,43,44]. Our analysis identified both drought-responsive MBS elements and ABA-responsive elements in the *OsAP4* promoter region (Figure 6A). In addition, phylogenetic analysis revealed that OsAP4 shares high evolutionary conservation with ASPG1 (Figure 1E), both exhibiting ubiquitous expression patterns and modulating ROS accumulation under drought stress, suggesting functional redundancy in their drought tolerance mechanisms [16]. Given that ASPG1 has been experimentally validated to confer drought resistance via the ABA signaling pathway, we hypothesized that OsAP4 might similarly participate in the hormonal stress responses. To test this, we quantified *OsAP4* expression levels under drought conditions with six phytohormone treatments to elucidate its potential crosstalk with hormone-mediated stress adaptation. Notably, ABA and CTK induced significant changes in *OsAP4* expression levels. Furthermore, ABA treatment also modulated the expression of catalase genes *OsCATA* and *OsCATC* (Figure 6B–D). These findings collectively suggest that the OsAP4-OsCATA/OsCATC module may participate in drought stress responses through ABA-mediated signaling pathways. However, given the complexity of hormonal crosstalk in stress responses, this proposed mechanism requires further experimental validation.

## 5. Conclusions

This study delineates a negative regulatory role of OsAP4 in drought stress adaptation. The OsAP4-OsCATA/CATC module modulates ROS scavenging to coordinate rice drought resilience, revealing novel molecular circuitry underlying stress responses. OsAP4 is a promising genetic target for enhancing drought resistance through precision genome editing using CRISPR/Cas9 technology in molecular breeding programs.

## Data Availability

Data will be made available upon request.

## Supplementary Materials

The following supporting information can be downloaded at: https://www.mdpi.com/article/10.3390/plants14142174/s1. Figure S1. Expression pattern of *OsAP4* under drought-mimicking stress conditions. (A) Expression of *OsAP4* in 0–12 h of mannitol-treated and (B) medium-free treated rice seedlings. Data are means from the TENOR database. Figure S2. Haplotype analysis of *OsAP4* associate with rice drought tolerance. (A) Haplotype information of the *OsAP4* genomic sequence of rice varieties in the MBKbase database. (B) Comparative analysis of drought tolerance grade of *Hap 3*, *Hap 1*, and *Hap 5*. Data are means ± SD (*n* ≥ 10); * *p* < 0.05, * *p* < 0.01 determined by *t*-test; the test was performed on samples between the *Hap 3* and *Hap 1/5*. Figure S3. Identification of *OsAP4* transgenic materials. (A) Expression of *OsAP4* in *OsAP4* overexpression lines. Data are means ± SD (*n* = 3); ** *p* < 0.01 determined by *t*-test; the test was performed on samples between the 9311 and OE lines. (B) Analysis of *OsAP4* protein frameshift mutations induced by target sequence variations in 9311 and *OsAP4* knockout lines. The red pentagram represents the termination of protein translation. Table S1. Primers used in this study.
