# Supplementary material for: The OsAP4-OsCATA/OsCATC Regulatory Module Orchestrates Drought Stress Adaptation in Rice Seedlings Through ROS Scavenging
Source: Plants (Basel). 2025 Jul 14;14(14):2174. doi: 10.3390/plants14142174 (PMC12298810; doi:10.3390/plants14142174)
Supplement: Supplementary file 1 [file plants-14-02174-s001.zip › Supplementary files 2025.7.12/Supplementary Figures.pdf]

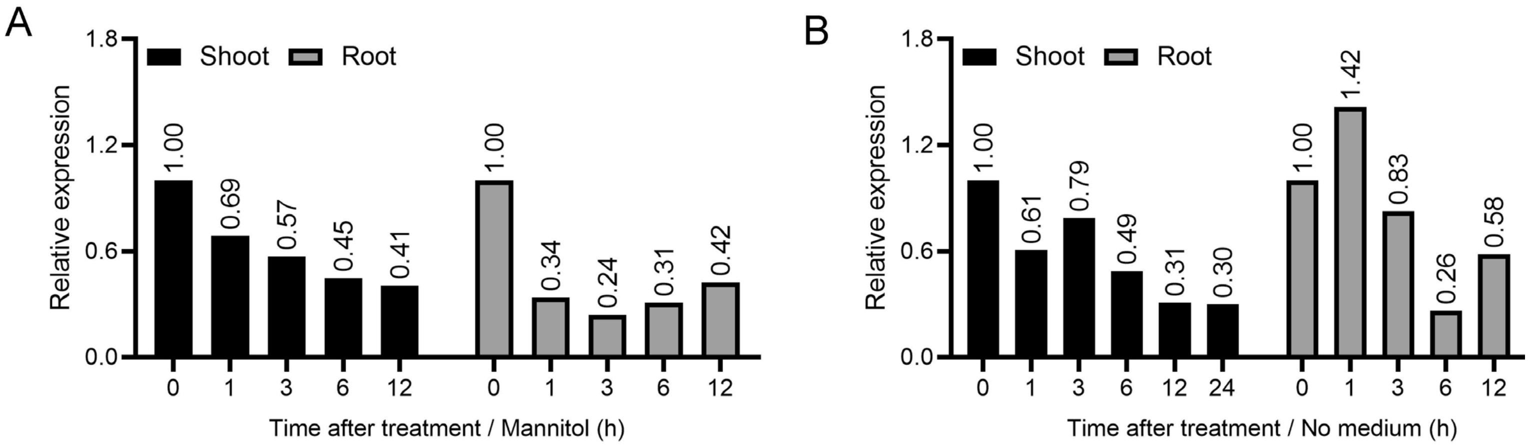

Figure S1. Expression pattern of *OsAP4* under drought-mimicking stress conditions. (A) Expression of *OsAP4* in 0–12 h of mannitol-treated and (B) medium-free treated rice seedlings. Data are means from the TENOR database.

A

Hap \ Position	2120384	2120392	2120412	2120416	2120446	2120507	2120510	2120529	2120574	2120581	2120674	2120688	2120695	2120732	2120764	2120821	2120833	2120845	2120866	2120908	2120998
Hap 1	G	G	G	G	C	G	T	C	C	A	A	T	C	G	C	C	A	T	C	C	T
Hap 2	G	A	C	A	C	C	C	A	T	G	G	C	G	A	T	G	C	C	G	T	G
Hap 3	G	G	G	G	C	G	T	C	C	A	G	T	C	G	C	C	A	T	C	C	T
Hap 4	A	G	G	G	T	G	T	C	C	A	G	T	C	G	C	C	A	T	C	C	T
Hap 5	G	G	G	G	T	G	T	C	C	A	G	T	C	G	C	C	A	T	C	C	T

Hap \ Position	2121040	2121346	2121373	2121385	2121391	2121412	2121415	2121574	2121587	2121641	2121772	2121859	2122019	2122036	2122063	2122088	2122112	2122141	2122151	2122337	No. of type
Hap 1	C	A	G	G	G	G	A	G	C	A	G	C	A	C	A	T	A	C	T	T	1811
Hap 2	T	C	C	C	T	C	T	C	A	G	C	G	T	G	C	G	C	G	C	C	484
Hap 3	C	A	G	G	G	G	A	G	C	A	G	C	T	C	A	T	A	C	T	T	475
Hap 4	C	A	G	G	G	G	A	G	C	A	G	C	A	C	A	T	A	C	T	T	312
Hap 5	C	A	G	G	G	G	A	G	C	A	G	C	T	C	A	T	A	C	T	T	250

B

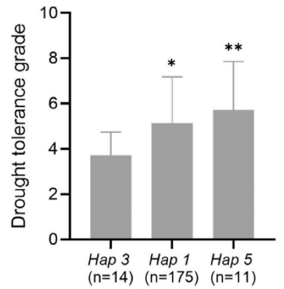

Figure S2. Haplotype analysis of *OsAP4* associate with rice drought tolerance. (A) Haplotype information of the *OsAP4* genomic sequence of rice varieties in the MBKbase database. (B) Comparative analysis of drought tolerance grade of *Hap 3*, *Hap 1*, and *Hap 5*. Data are means ± SD ($n \geq 10$); $^{*}P < 0.05$, $^{**}P < 0.01$ determined by *t*-test; the test was performed on samples between the *Hap 3* and *Hap 1*/*5*.

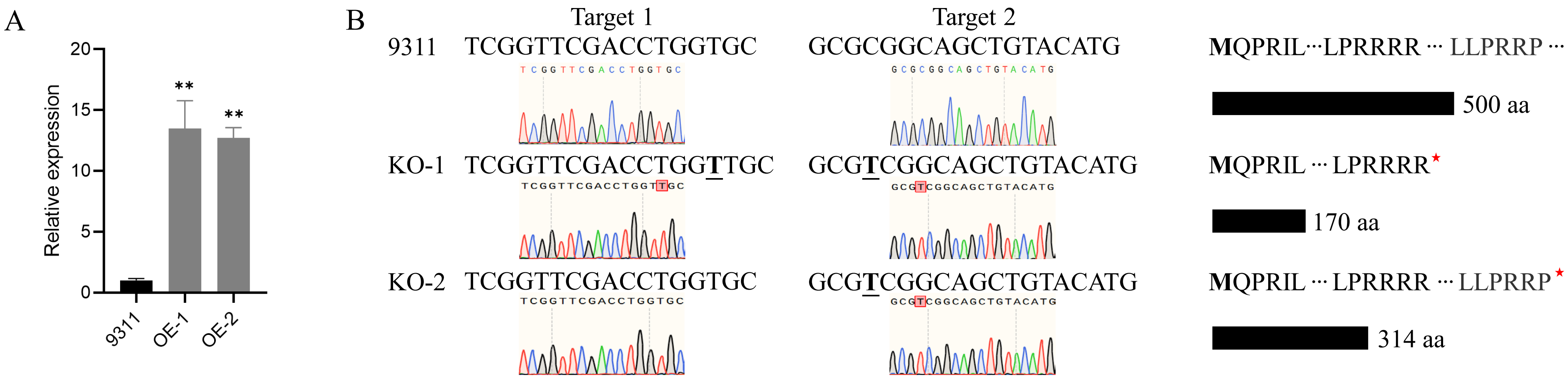

Figure S3. Identification of *OsAP4* transgenic materials. (A) Expression of *OsAP4* in *OsAP4* overexpression lines. Data are means ± SD (n = 3); $^{**}P < 0.01$ determined by t-test; the test was performed on samples between the 9311 and OE lines. (B) Analysis of OsAP4 protein frameshift mutations induced by target sequence variations in 9311 and *OsAP4* knockout lines. The red pentagram represents the termination of protein translation.
